# Transpulmonary LOX-1 Levels Are Predictive of Acute Respiratory Distress Syndrome After Cardiac Surgery: A Proof-of-Concept Study

**DOI:** 10.3390/biomedicines13040800

**Published:** 2025-03-26

**Authors:** Benjamin Deniau, Pierre-Olivier Ludes, Pamela Khalifeh-Ballan, Luc Fenninger, Michel Kindo, Olivier Collange, Bernard Geny, Eric Noll, Fériel Azibani, Alexandre Mebazaa, Julien Pottecher

**Affiliations:** 1 Department of Anaesthesia, Burn and Critical Care, University Hospitals Saint-Louis-Lariboisière, AP-HP, 75010 Paris, France; benjamin.deniau@aphp.fr (B.D.); alexandre.mebazaa@aphp.fr (A.M.); 2 UMR-S 942, INSERM, MASCOT, Paris University, 75018 Paris, France; feriel.azibani@inserm.fr; 3 Department of Medicine, Paris Cité University, 75006 Paris, France; 4 FHU PROMICE, Hôpital Lariboisière 2, rue Ambroise Paré, 75475 Paris, CEDEX 10, France; 5 INI CRCT, CHRU Brabois, 54500 Vandoeuvre les Nancy, France; 6 Department of Anaesthesiology Critical Care and Perioperative Medicine, Hautepierre Hospital, Strasbourg University Hospital, 67000 Strasbourg, Francepamela@ballans.eu (P.K.-B.); luc.fenninger@chru-strasbourg.fr (L.F.); eric.noll@chru-strasbourg.fr (E.N.); 7 Department of CardioVascular Surgery, Nouvel Hôpital Civil, Strasbourg University Hospital, 67000 Strasbourg, France; michel.kindo@chru-strasbourg.fr; 8 UR 3072, « Mitochondrie, Stress Oxydant et Protection Musculaire », FMTS, FHU Omicare, Faculty of Medicine, Midwifery and Health Sciences, Strasbourg University, 67081 Strasbourg, France; olivier.collange@chru-strasbourg.fr (O.C.); bernard.geny@chru-strasbourg.fr (B.G.); 9 Department of Anaesthesiology Critical Care and Perioperative Medicine, Nouvel Hôpital Civil, Strasbourg University Hospital, 67000 Strasbourg, France; 10 Service de Physiologie et d’Explorations Fonctionnelles, Nouvel Hôpital Civil, Strasbourg University Hospital, 67000 Strasbourg, France

**Keywords:** acute respiratory distress syndrome, LOX-1, large-scale proteomics, outcome, prognosis, cardiac surgery, OLINK assay, biomarker

## Abstract

**Background/Objectives:** Acute respiratory distress syndrome (ARDS) is a life-threatening condition that frequently complicates high-risk cardiac surgery. We evaluated the circulating levels and transpulmonary gradient of intracellular proteins in patients at risk of developing ARDS after cardiac surgery using large scale-proteomics. **Methods:** We enrolled sixteen patients undergoing high-risk cardiac surgery, followed by planned ICU admission. Circulating levels of intracellular proteins were measured at the onset of the surgical procedure, at ICU admission (H0), and 24 h (H24) after surgery in blood samples simultaneously drawn from both the pulmonary artery and the left atrium. The primary endpoint was the occurrence of ARDS between ICU admission and the subsequent 48 h. **Results:** Among the studied proteins, the levels of intracellular lectin-like oxidized low-density lipoprotein receptor-1 (LOX-1) were higher at H24 in the pulmonary artery in patients who developed ARDS (6.96; 95% CI [6.83–7.23]) compared to patients who did not (6.48; 95% CI [6.27–6.66]), *p*-value = 0.016. The transpulmonary gradient of intracellular LOX-1 levels was not significantly different between ARDS and non-ARDS patients at H0 but it was more negative at H24 in ARDS (−0.23; 95% CI [−0.27, −0.14]) than in non-ARDS patients (0.03; 95% CI [−0.14, 0.32]; *p*-value= 0.031), with a hazard ratio HR = 0.39 (95% CI [0.18–0.86]); *p*-value= 0.035. The area under the ROC curve of H24 LOX-1 transpulmonary gradient to predict ARDS occurrence was 0.83 (95% CI [0.62–1.00]). **Conclusions:** The transpulmonary gradient of intracellular LOX-1 levels was negatively associated with the occurrence of ARDS within the first 48 h after high-risk cardiac surgery, suggesting that lung trapping of LOX-1 may be linked to postoperative ARDS.

## 1. Introduction

Acute respiratory distress syndrome (ARDS) is a frequent and life-threatening condition, occurring in 10% of patients admitted to the intensive care unit (ICU) and in 23% of mechanically ventilated patients [1]. Acute respiratory distress syndrome is associated with poor outcomes [1]. Its definition, based on Berlin and Global criteria [2], includes an onset within 7 days of a known clinical insult (or new or worsening respiratory symptoms), bilateral opacities on chest imaging, a decreased PaO_2_/FiO_2_ ratio < 300, and a minimum PEEP setting or CPAP [3,4]. The pathophysiology of ARDS is not completely understood, but it likely includes capillary congestion, atelectasis, and intra-alveolar hemorrhage, leading to diffuse alveolar damage in the early stage. Alveolar edema later gives way to hyaline membrane formation, epithelial cell hyperplasia, and fibrosis [5]. Causes and risk factors are diverse and include pneumonia and non-pulmonary sepsis, as well as trauma and major surgery [6]. As a matter of fact, ARDS is observed after cardiac surgery with a variable incidence, exceeding 8% after extensive procedures [7]. The pathophysiology of ARDS following cardiac surgery remains incompletely understood, but it may result from a combination of preoperative risk factors, tissue attrition, cardiopulmonary bypass (CBP), and mechanical ventilation with the transfusion of blood products, which is sometimes responsible for transfusion-related acute lung injury [8]. The main risk factors for ARDS following cardiothoracic surgery include the use of cardiopulmonary bypass, long bypass durations, ischemia–reperfusion episodes triggering reactive oxygen species generation, lung transplantation (with primary graft dysfunction), transfusion-related acute lung injury, and drug toxicity (notably amiodarone). Although ARDS diagnosis has been simplified thanks to the Berlin and Global criteria, its prevention and prediction remain major challenges for ICU physicians, anesthesiologists, and cardiac surgeons [9,10]. Biomarkers could fill this gap and play a central role in the early identification and prognosis of ARDS [11]. For instance, von Willebrand Factor, angiopoietin 2, and interleukine-8 (among others) have been found to be elevated and associated with ARDS outcomes [11,12,13]. Low-density lipoprotein receptor-1 (LOX-1) is a scavenger receptor expressed on several cell types, including endothelial cells in many vascular-rich organs (the aorta, thoracic arteries, lungs, brain, liver, and placenta) [14,15], but also on polymorphonuclear cells, macrophages, platelets, and vascular smooth-muscle cells [16]. Several studies have shown that LOX-1 is involved in inflammatory processes, including atherosclerosis, cell death, and inflammatory cytokine production [17,18]. Recently, Korkmaz et al. demonstrated that LOX-1 accumulates in the lungs of patients with ARDS, confirmed this finding in pneumonia-induced ARDS in mice, and suggested a potential protective role for this receptor [14]. However, the precise role of LOX-1 in the pathophysiology and development of ARDS remains unknown. Moreover, current ARDS prediction models are primarily designed for general ICU populations and do not adequately account for cardiac surgery-specific risk factors such as cardiopulmonary bypass, fluid shifts, transfusions, and intraoperative ventilatory strategies. Existing studies on postoperative ARDS often rely on small cohorts, lack external validation, or are not easily applicable in clinical practice. A dedicated prediction model incorporating these specific variables and biomarkers is needed to improve early identification and management of at-risk patients. Thus, we still lack sensitive and specific biomarkers for predicting postoperative ARDS in cardiac surgery patients, and the primary site of biomarker release into the systemic circulation remains unclear. Indeed, identifying the release site (either systemic circulation or lungs) of ARDS-triggering and biologically active biomarkers would provide clues to develop targeted strategies. We leveraged the routine invasive hemodynamic monitoring performed in selected high-risk cardiac surgery patients, which allowed us to simultaneously sample blood from the pulmonary artery and the left atrium. This approach enabled us to investigate the association between the transpulmonary gradient of biomarker levels and the occurrence of postoperative ARDS. Using large-scale proteomics, the aim of this study was to assess the ability of a biomarker panel to predict the occurrence of ARDS within the first 48 postoperative hours in patients undergoing high-risk cardiac surgery.

## 2. Materials and Methods

### 2.1. Study Design and Population

This was an interventional, prospective, and monocentric study performed at Hôpitaux Universitaires de Strasbourg, France, between November 2013 and October 2015. The recruitment period started on 12 November 2013 and ended on 25 October 2015. All procedures were performed in compliance with relevant laws and institutional guidelines. This study was conducted in accordance with the Declaration of Helsinki and was approved by the local research ethics committee (Comité de Protection des Personnes, approval # IDRCB 2013-A00462-43, on 14 May 2013). The privacy rights of all subjects were respected, and written informed consent was obtained from all patients before study inclusion. The study protocol was registered on ClinicalTrials.gov before study initiation (NCT01723930, registered 6 November 2012).

The inclusion criteria were as follows: age > 18 years, cardiac surgery followed by planned ICU admission for postoperative observation, predicted high risk for postoperative respiratory compromise according to Kor et al.’s criteria [14] (high-risk cardiac surgery, diabetes mellitus, chronic obstructive pulmonary disease, gastroesophageal reflux disease, and alcohol abuse), planned insertion of left atrial and pulmonary artery catheters, and planned duration of cardiopulmonary bypass (CPB) exceeding 120 min. As published by Kor et al., a Surgical Lung Injury Prediction (SLIP) score ≥ 22 points is associated with a much higher risk of postoperative ARDS [19]. Non-inclusion criteria were emergent surgery (making informed consent impossible), acute heart failure, preoperative mechanical ventilation, latex allergy, guardianship, pregnancy, and breastfeeding. The criteria for high-risk cardiac surgery in our cohort included combined cardiac surgery (valve replacement associated with coronary artery bypass graft surgery), three-vessel coronary artery bypass graft surgery, double or triple valve replacement surgery, and redux cardiac surgery (either valve or coronary artery bypass graft).

### 2.2. Measurements

**Figure 1:**
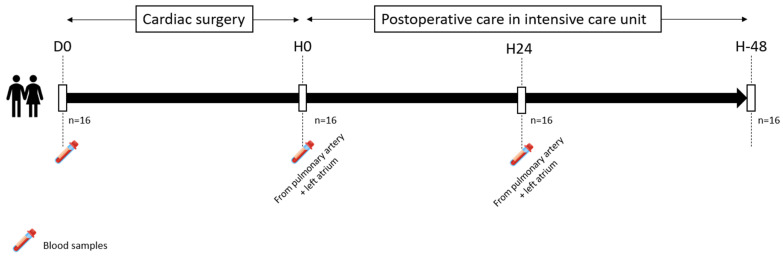
Protocol of the study, from day 0 (surgery) to H-48 (48 h after surgery). (D0 = day 0: onset of the surgical procedure).

The study protocol is summarized in Figure 1. Briefly, on the day of cardiac surgery, the included patients were anesthetized under general anesthesia according to local practices. Patients were sampled at the onset of the surgical procedure (day 0 sample, D0) upon ICU admission post-surgery (hour 0, H0), and after 24 h of ICU care (hour sample 24, H24) (Figure 1). At H0 and H24, blood was simultaneously sampled from two different locations along the circulatory pathway surrounding the lungs: the main pulmonary artery using a pulmonary artery catheter (PAC samples) and through a surgically inserted left atrial catheter (left atrium (LA) samples). At the time this study was conducted, LA catheters were routinely inserted in all cardiac surgery patients to monitor left atrial pressure and estimate left ventricular preload. They were typically removed on postoperative day 2, along with the mediastinal drains. Pulmonary artery catheters were inserted in selected patients when the attending anesthesiologist considered that continuous monitoring of cardiac output and pulmonary artery pressure was required to steer intraoperative and postoperative management. The difference in plasma biomarker concentration between the left atrium and the pulmonary artery (LA-PA) determined whether the biomarker was released by the lungs into the circulation (when this difference was positive) or captured by the lungs (when this difference was negative). Using a similar methodology, Bendjelid et al. demonstrated lactate release after hypothermic CPB [20]. We refined this process and addressed their acknowledged limitation of potential systemic biomarker release by blood sampling from the left atrium—exiting the lung circulation—rather than a more distal site, such as the radial artery.

### 2.3. Data Collection

Clinical variables (including age, sex, body mass index, smoking status, medical history, treatments, and preoperative laboratory values), details of the surgical procedure (type, bypass duration, and duration of aortic cross-clamping), and key oxygenation parameters (PaO_2_/FiO_2_ ratio and alveolo-arterial oxygenation difference [AaDO_2_] at H0 and H24) were electronically recorded. Cardiac output, pulmonary artery pressure, pulmonary artery wedge pressure, pulmonary capillary pressure, left atrial pressure, and pulmonary vascular resistance (including arterial and venous contribution) were determined using conventional formulas and recommended maneuvers [21] at each time point as long as pulmonary artery and left atrial catheters were in place. EDTA plasma samples were centrifuged for 10 min at 3000 rpm, and the supernatant was frozen and stored at −80 °C.

### 2.4. Proteomics Assay

Measurements of intracellular protein levels were performed using the Olink^®^ assay (Proseek^®^ Multiplex Cardiovascular II and Cardiovascular III panels, Olink Proteomics, Boston, MA, USA). The Olink immunoassay uses a proximity extension technology with dual-recognition DNA-coupled readout to provide normalized protein expression values [22,23]. The Olink^®^ Cardiovascular II and III panels sample 184 proteins and provide log2 normalized protein expression values with relative quantification.

### 2.5. Endpoints

The primary endpoint was the occurrence of ARDS, defined according to the Berlin definition as bilateral opacities consistent with pulmonary edema on chest X-ray or computed tomography, not fully explained by cardiac failure or fluid overload, with PaO_2_/FiO_2_ ratio < 300 and with a minimum positive end expiratory pressure or continuous positive airway pressure of 5 cm of water [1] between ICU admission and H48. As our cardiosurgical patients were equipped with a pulmonary artery catheter and left atrial pressure catheter and monitored with repeated echocardiography (transthoracic and transesophageal), we considered that pulmonary edema was not fully explained by cardiac failure and fluid overload in the following cases:

○ The transmural left atrial pressure or the transmural pulmonary artery occlusion pressure were inferior or equal to 18 mmHg.
○ The ratio of early diastolic mitral inflow velocity to early diastolic mitral annulus velocity (E/e’ ratio), estimated using tissue Doppler imaging, was inferior or equal to 14.
○ The left ventricular ejection fraction had not decreased by more than 15% compared to a previous assessment when the patient was not hypoxemic.

### 2.6. Bioinformatics Analysis

Proteins measured by Olink^®^ assay are known to be involved in several biological domains. We performed an extensive search of the biomedical literature to identify effects of proteins and potential involvement in the pathophysiology of ARDS. A volcano plot analysis was chosen to identify candidate proteins whose expression would be associated with postoperative ARDS. We identified differentially expressed proteins as those fulfilling the two following independent criteria: (i) |log2 fold change| > log2(1.1), corresponding to a ≥10% increase from baseline (log2 fold change > 0.1375), and (ii) a false discovery rate q-value (FDRq) < 1%, which applies a Benjamini–Hochberg correction to the raw *p*-value, in order to minimize inflation of a false-positive error rate due to multiplicity of comparisons [23,24].

### 2.7. Statistical Analysis

The normality of data distribution was tested by a Kolmogorov–Smirnov normality test. Continuous variables were expressed as median and interquartile ranges (IQRs) and were compared with the Mann–Whitney U test. Categorical variables were expressed as counts and percentages and were compared with Fisher’s exact test or the chi square test, as appropriate. Potential association between biomarkers and between hemodynamic variables and biomarkers were graphically assessed using correlograms before formal statistical tests for association were performed, when considered relevant. Odds ratios (ORs) or hazard ratios (HRs) are presented with their 95% confidence interval (CI). Statistical analysis was conducted using R software (R Core Team, version 4.4.3 (2025)). R Foundation for Statistical Computing, Vienna, Austria) and figures were produced using ggplot2 package [25]. A *p*-value < 0.05 was considered statistically significant.

## 3. Results

### 3.1. Baseline Characteristics

Between November 2013 and October 2015, 16 patients were enrolled in this study. The patients’ characteristics are summarized in Table 1. Briefly, the cohort was mainly composed of men (10 (62%)) with a median age of 74 (66–80) years old. Concerning medical history, diabetes mellitus, hypertension, and dyslipidemia were the most common diseases, in seven (44%), twelve (75%) and eight (50%) patients, respectively; four (25%) patients had previously experienced valvular replacement. Eleven (69%), ten (62%), and eight (50%) patients were daily treated with diuretics, beta-blockers, and angiotensin-converting enzyme inhibitors, respectively (Table 1). Median preoperative left ventricular ejection fraction was 55% (IQR 36–61%). Surgical procedures were mainly valve replacement (9 (56%)) and valve replacement combined with coronary artery bypass graft (6 (38%)). Median bypass duration was 129 (IQR 106–178) minutes, with aortic cross-clamping of 93 (IQR 74–119) minutes.

### 3.2. Occurrence of ARDS After Cardiac Surgery

**Figure 2:**
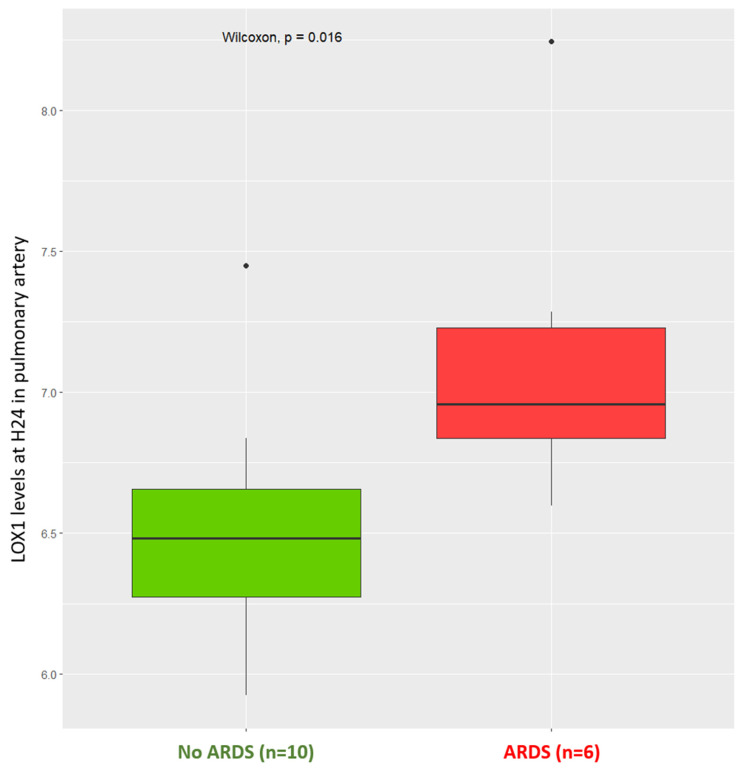
Levels of intracellular LOX-1 at H24 after cardiac surgery measured in the pulmonary artery in patients who developed acute distress respiratory syndrome (red) and in those who did not (green). (ARDS: acute respiratory distress syndrome).

**Figure 3:**
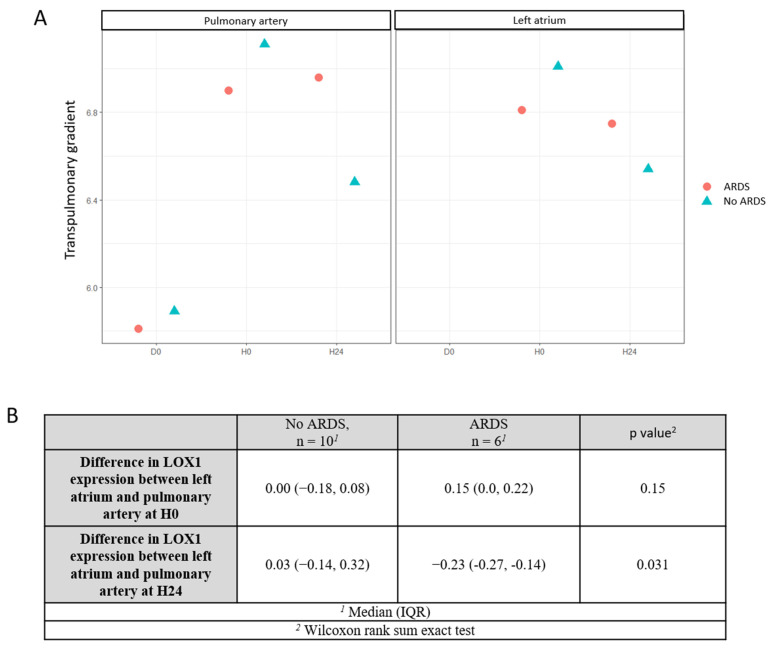
(**A**) Transpulmonary gradient (difference between levels between left atrium and pulmonary artery) of LOX-1 levels at H0 and H24 in ARDS (red dot) and non-ARDS patients (green triangle), (**B**) Difference in LOX-1 expression between left atrium and pulmonary artery at H0 and H24. (ARDS: acute respiratory distress syndrome).

**Figure 4:**
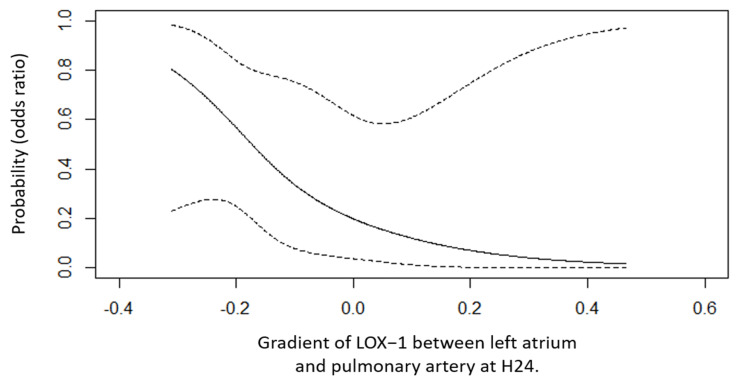
Spline plot depicting the probability of developing acute respiratory distress syndrome after cardiac surgery according to the gradient of LOX-1 between the left atrium and pulmonary artery at H24.

Among the 16 patients enrolled in the study, 6 (38%) developed ARDS between ICU admission (H0) and H48. In every case, respiratory failure was not explained by cardiac failure or fluid overload (LAP measurement and echocardiography allowed for an objective assessment of left ventricular telediastolic pressure to exclude hydrostatic edema as the only cause for respiratory failure). Volcano plots of levels of intracellular proteins measured by the Olink^®^ assay at H0 and H24 after cardiac surgery in pulmonary artery and left atrium are depicted in the Supplemental Data, Figure S1. We identified several candidate intracellular proteins differentially expressed between patients who developed ARDS after cardiac surgery and those who did not, including lectin-like oxidized low-density lipoprotein receptor-1 (LOX-1) (see Supplemental Data, Figure S1). Levels of intracellular LOX-1 in the pulmonary artery and left atrium in patients after cardiac surgery pm D0 and H0 were not different between patients who developed ARDS and patients who did not (Table 2), but we observed higher H24 levels of intracellular LOX-1 in the pulmonary artery in patients who developed ARDS (6.96; 95% CI [6.83–7.23]) when compared to patients who did not (6.48; 95% CI [6.27–6.66]), with *p*-value = 0.016 (Figure 2). The transpulmonary gradient of LOX-1 levels was non-significantly different between ARDS and non-ARDS patients at H0 but it was more negative at H24 in patients who developed ARDS (−0.23; 95% CI [−0.27, −0.14]) than in those who did not (0.03; 95% CI [−0.14, 0.32]; *p*-value = 0.031), with a HR = 0.39 (95% CI 0.18–0.86), *p*-value = 0.035 (Figure 3). As depicted in Figure 4, the more negative the value of the transpulmonary gradient of LOX-1 plasma levels, the higher the risk of developing postoperative ARDS. Finally, the area under the receiver operating characteristic (ROC) curve (AUC) of H24 LOX-1 transpulmonary gradient plasma expression to predict ARDS occurrence was 0.83 (95% CI [0.62–1.00]). The venous contribution of pulmonary vascular resistances and the difference between capillary and left atrial pressures were associated with the alveolo-arterial oxygenation difference (Spearman’s rank correlation coefficient r = 0.5, *p*-value < 0.05). It is worth mentioning that the correlogram analysis did not find any statistical or meaningful association between the hemodynamic variables and the biomarkers assayed (Spearman’s rank correlation coefficient r < 0.5, *p*-value > 0.05) (Supplementary Figure S2A–H).

## 4. Discussion

Our results established that, after cardiac surgery (mainly valve replacement and/or coronary artery bypass graft), the intracellular concentration levels of LOX-1, a scavenger receptor involved in inflammatory processes, were increased during the first 24 h after surgery in the pulmonary artery of patients who developed ARDS. In addition, we observed that the more negative the value of the transpulmonary gradient of LOX-1 levels, the higher the risk of developing postoperative ARDS within 48 h of surgery.

Acute respiratory distress syndrome is a common condition with various etiologies, mainly observed in severely ill patients and associated with high morbidity and mortality [6]. The Berlin definition and its recent update [2] codified the clinical definition of this syndrome in different stages according to the value of PaO_2_/FiO_2_ ratio [3,4]. Recent research described a large heterogeneity of types of ARDS [26,27], reflecting its complex pathophysiology, in which precision medicine could provide clues for an effective therapy.

To date, the pathophysiology of ARDS is not completely understood, and it involves several pathways [6] which may be differentially activated across the multiple phenotypes, including postoperative ARDS after cardiac surgery. Local and systemic inflammation are prominent features in ARDS, leading to lung injury [28]. Several varieties of mediators, including reactive oxygen species, proteases, and pro-inflammatory mediators (e.g., prostaglandins and leukotrienes), have been observed in the early course of ARDS in lungs [28,29]. A central role of the activation NRLP3 inflammasome initiating a local release interleukin-1-β and interleukin-18 has also been described [30], testifying to the large complexity of the pathophysiology of ARDS.

It is demonstrated that the progression of lung damage to ARDS depends on integrated contributions of host systems, reducing pathogen burden (in case of infectious etiology) and limiting tissue injuries [17,18], which lead to ARDS [18]. Low-density lipoprotein receptor-1 (LOX-1) is a scavenger receptor (50-kDA transmembrane glycoprotein) expressed by various cell types and playing a central role in inflammatory processes [18,31,32]. Recent findings suggest an accumulation in lungs of patients with ARDS, confirmed by murine model of pneumonia [18]. However, its precise role is currently unknown and needs to be studied. LOX-1 contributes to endothelial dysfunction and apoptosis. Recently, Korkmaz et al. demonstrated the central role of LOX-1 in the development of sepsis-induced ARDS by pneumonia in human and mice [18]. Indeed, authors demonstrated that LOX-1 was accumulating in the lungs of patients with ARDS. Based on an experimental model of bacterial pneumonia, they also observed a protective role of LOX-1 in the pulmonary airspaces, mainly expressed by alveolar macrophages and recruited neutrophils, limiting proteinaceous and edema inflammation [14]. These observations are in opposition to previous studies, where LOX-1 was involved in vascular inflammation, atherosclerosis [18], and the production of reactive oxygen species and inflammatory cytokines [32]. In our study, we observed a negative association between the transpulmonary gradient of the intracellular concentration of LOX-1 and the development of ARDS during the first 48 h after cardiac surgery. Although our results are based on immunoassay and not on the concentration and/or activity of the protein, the negative gradient of intracellular LOX-1 levels between the left atrium and pulmonary artery at H24 in patients developing postoperative ARDS could testify to an increased lung capture of the protein. This is in line with the main results of Korkmaz et al. [14]. As macrophages and neutrophils were the two prominent sites of LOX-1 expression in the pulmonary airspaces in Korkmaz’s findings, the 24 h delay we observed between the end of surgery and negative transpulmonary LOX-1 levels is consistent with similar sites of expression.

Using a methodology similar to ours but in a larger cohort of patients, Wang et al. were able to identify three proteins which were differentially expressed after cardiopulmonary bypass in patients who subsequently developed ARDS: thioredoxin domain containing 5 (TXNDC5), cathepsin L (CTSL), and NPC intracellular cholesterol transporter 2 (NPC2). It is interesting to emphasize that both LOX-1 and NPC2 are glycoproteins dealing with cholesterol transport [33].

Comparing LOX-1 expression on the surface of polymorphonuclear neutrophils (PMN) assayed simultaneously in blood (cPMN) and tracheal aspirates (tPMN) in ARDS and control patients, Kraus et al. showed that LOX-1 expression on tPMN was largely increased, while it remained stable in cPMN, reinforcing our findings of a negative transpulmonary gradient and lung trapping of LOX-1 in ARDS patients [34]. In patients suffering from viral (COVID-19) or bacterial sepsis, Coudereau et al. explored the phenotype, morphology, and immunosuppressive functions of whole-blood LOX-1+ cells. These LOX-1+ cells were polymorphonuclear myeloid-derived suppressor cells with immunosuppressive properties (repression of T-cell response ascertained by reduced IFN-γ production); they peaked some days (3 to 9 days) after intensive care unit admission and significantly increased within the first three days in COVID-19 patients who developed ARDS within this time frame [35]. In ARDS complicating cardiac surgery, increased LOX-1 expression and lung sequestration may participate both in the dampening of tissue inflammation and in the development of persistent inflammation, immunosuppression, and catabolism syndrome (PICS).

In the light of our results, several questions remain unanswered. To date, it is not clear whether the negative transpulmonary gradient of intracellular LOX-1 levels after cardiac surgery, as observed in our study, is either (i) involved in inflammatory processes, leading to ARDS development or (ii) contributes to a dampening effect on local inflammation signaling or (iii) remains an inactive bystander of the inflammatory process. The design of our clinical study precludes any supplemental mechanistic approach, which may be addressed in experimental animal studies. Based on the previous results obtained by Korkmaz et al. [14], including experimental pneumonia in LOX-1 KO mice and anti-LOX-1 antibodies, and the long-standing description of neutrophil lung sequestration during ARDS [36], we may speculate that neutrophils and macrophages were captured in the lung parenchyma where they expressed LOX-1 to promote M2-like macrophage polarization and tissue repair in postoperative ARDS patients. This hypothesis should be further validated.

To our knowledge, this is the first study describing a potential association between a negative transpulmonary gradient of intracellular LOX-1 levels 24 h after cardiac surgery and the development of postoperative ARDS. We found a negative association between the transpulmonary levels of intracellular LOX-1 after cardiac surgery and the development of ARDS during the first 48 h, with a potentially fair predictive performance which should be confirmed on a larger scale. While preliminary, these new findings appear promising and give us the opportunity to make hypotheses concerning the pathophysiology of postoperative ARDS for future research. Indeed, future research should focus on validating the proposed ARDS prediction model in larger multicenter cohorts to ensure its generalizability and clinical applicability. Additionally, mechanistic studies on LOX-1 in lung injury are needed to elucidate its role in ARDS pathophysiology, potentially identifying new therapeutic targets. Combining predictive modeling with biomarker-driven approaches could further enhance early risk stratification and personalized management strategies. Future research should also prioritize large-scale, multicenter validation studies to confirm the predictive value of LOX-1 and refine ARDS risk stratification models in cardiac surgery patients. Prospective clinical trials integrating LOX-1 measurements into perioperative risk assessment could help determine its utility in guiding early therapeutic interventions. Additionally, mechanistic research using preclinical animal models is essential to elucidate the role of LOX-1 in lung endothelial dysfunction, inflammation, and permeability changes leading to ARDS. Experimental studies could also explore potential therapeutic strategies targeting LOX-1 to mitigate lung injury. A translational approach, combining clinical validation with fundamental research, may ultimately lead to novel precision medicine strategies for ARDS prevention and management in high-risk surgical populations.

Our study has some limitations. First, the number of patients enrolled is low, limiting the interpretation of results and external validity of this work. These were high-risk cardiosurgical patients, selected on stringent criteria and invasively monitored, limiting recruitment. In the same way, while the ROC AUC of 0.83 suggests promising predictive performance, the wide confidence interval reflects the small sample size and inherent variability, suggesting that the results should be interpreted with caution. These proof-of-concept results need to be confirmed by further studies. Second, the Olink^®^ assay provides the intracellular concentration levels of a protein of interest and does not correspond to a concentration and/or a protein activity. Therefore, no extrapolation of our results and no comparison with other studies (i.e., studying concentration and activity) can be made. Third, the soluble form of LOX-1 (as measured by the Olink^®^ assay) may have biological actions different from its membrane-bound counterpart, which was not addressed in our study. Fourth, the Olink^®^ assay is not in use in daily practice, which limits its integration in routine clinical care for prediction purposes.

## 5. Conclusions

The findings of this study showed that the transpulmonary gradient of intracellular LOX-1 was negative in patients developing ARDS during the first 48 h after cardiac surgery, suggesting increased pulmonary uptake of LOX-1. This observation aligns with previous studies highlighting the role of LOX-1 in inflammatory and endothelial processes, but its precise involvement in ARDS pathophysiology remains to be elucidated. While our study provides a novel perspective on LOX-1 dynamics in the postoperative setting, the small sample size limits the generalizability of our findings and warrants cautious interpretation. Our proof-of-concept results highlight the potential of LOX-1 as a biomarker for early ARDS detection in high-risk surgical patients. Mechanistic studies in experimental models should investigate whether LOX-1 plays a causative or compensatory role in lung injury and whether its modulation could have therapeutic implications. These findings could open new avenues for precision medicine approaches in perioperative care.

## Figures and Tables

**Table 1:** Characteristics of the patients.

Variables	Overall (n = 16) *	No ARDS (n = 10) *	ARDS (n = 6) *	*p*-Value ^†^
Patient characteristics				
Age, years (IQR)	74 (66–80)	74 (68–77)	78 (62–84)	0.6
Female, n (%)	6 (38)	4 (40)	2 (33)	>0.9
Body mass index, kg/m^2^ (IQR)	23.8 (22.4–28.6)	23.8 (22.7–26.6)	25.8 (21.8–29.2)	>0.9
Medical history, n (%)				
Smoking	1 (6.3)	0 (0)	1 (17)	0.4
Diabetes mellitus	7 (44)	5 (50)	2 (33)	0.6
Hypertension	12 (75)	7 (70)	5 (83)	>0.9
Dyslipidemia	8 (50)	5 (50)	3 (50)	>0.9
Atrial fibrillation	7 (44)	4 (40)	3 (50)	>0.9
Previous valvular replacement	4 (25)	2 (20)	2 (33)	0.6
Chronic obstruction pulmonary disease	5 (31)	3 (30)	2 (33)	>0.9
Medication, n (%)				
Beta-blockers	10 (63)	6 (60)	4 (67)	>0.9
Angiotensin-converting enzyme inhibitors	8 (50)	7 (70)	1 (17)	0.12
Angiotensin receptor blockers	4 (25)	2 (20)	2 (33)	0.6
Diuretics	11 (69)	8 (80)	3 (50)	0.3
Preoperative biology and transthoracic echocardiography				
Plasmatic creatinine, µmol/L (IQR)	73 (65–88)	71 (66–82)	80 (61–90)	>0.9
Ejection fraction, % (IQR)	55 (36–61)	55 (35–60)	53 (47–60)	0.7
Surgery				
Surgical procedure				>0.9
Coronary artery bypass, n (%)	1 (6.3)	1 (10)	0 (0)	
Coronary artery bypass with valve placement, n (%)	6 (38)	4 (40)	2 (33)	
Valve replacement, n (%)	9 (56)	5 (50)	4 (67)	
Bypass duration, min (IQR)	129 (106–178)	140 (120–185)	111 (105–130)	0.2
Aortic cross clamping, min (IQR)	93 (74–119)	104 (88–126)	77 (73–95)	0.4

* Data are shown as median with interquartile ranges or n (%). ^†^ Continuous variables were compared with the Mann–Whitney U test; categorical variables were compared with Fisher’s exact test or the chi square test, as appropriate. Abbreviations: ARDS, acute respiratory distress syndrome; IQR, interquartile range.

**Table 2:** LOX-1 expression within the first 48 h after cardiac surgery in patients who developed ARDS and in those who did not.

Time Point	Site of Sampling	No ARDS	ARDS	Difference	95% CI	*p*-Value
D0 * ^†^	Pulmonary artery	5.89 (5.81, 6.16)	5.81 (5.75, 5.90)	−0.20	−1.3, 0.85	0.7
H0 *	Pulmonary artery	7.11 (6.90, 7.23)	6.90 (6.78, 7.64)	−0.05	−0.94, 0.84	0.9
H0 *	Left atrium	7.01 (6.85, 7.38)	6.81 (6.64, 7.50)	0.05	−0.67, 0.78	0.9
H24 *	Pulmonary artery	6.48 (6.27, 6.66)	6.96 (6.83, 7.23)	−0.65	−1.3, −0.02	0.016
H24 *	Left atrium	6.54 (6.27, 6.81)	6.75 (6.64, 6.97)	−0.36	−1.0, 0.26	0.2

* D0 corresponds to the onset of the surgical procedure, H0 to ICU admission, and H24 to 24 h after ICU admission. ^†^ At D0, the left atrial catheter had not been inserted yet, so the left atrial blood sample could not be drawn at this time point. Abbreviations: ARDS, acute respiratory distress syndrome; CI, confidence interval.

## Data Availability

The original contributions presented in this study are included in the main text and Supplementary Material. Further inquiries can be directed to the corresponding author.

## Supplementary Materials

The following supporting information can be downloaded at https://www.mdpi.com/article/10.3390/biomedicines13040800/s1, Figure S1: Volcano plots of levels of intracellular proteins measured by the Olink^®^ assay. (A), H0 in the pulmonary artery; (B), H0 in the left atrium; (C), H24 in the pulmonary artery; and (D), H24 in the left atrium. Figure S2: Correlograms depicting the relationship between gradient biomarkers and hemodynamic variables between the left atrial and pulmonary artery at H0 and the left atrial and pulmonary artery at H24.

## Abbreviations

The following abbreviations are used in this manuscript:

AaDO2	Alveolo-arterial oxygenation difference
ARDS	Acute respiratory distress syndrome
AUC	Area under the curve
CI	Confidence interval
CPB	Cardiopulmonary bypass
D0	Day 0
FDRq	False discovery rate q-value
H0	Hour 0
H24	Hour 24
HR	Hazard ratios
ICU	Intensive care unit
IQR	Interquartile ranges
LA	Left atrium
LOX-1	Lectin-like oxidized low-density lipoprotein receptor-1
OR	Odds ratios
PA	Pulmonary artery
PAC	Pulmonary artery catheter
ROC	Receiver operating characteristic curve
SLIP	Surgical Lung Injury Prediction
