# Supplementary material for: Transpulmonary LOX-1 Levels Are Predictive of Acute Respiratory Distress Syndrome After Cardiac Surgery: A Proof-of-Concept Study
Source: Biomedicines. 2025 Mar 26;13(4):800. doi: 10.3390/biomedicines13040800 (PMC12024757; doi:10.3390/biomedicines13040800)
Supplement: Supplementary file 1 [file biomedicines-13-00800-s001.zip › Supplementary Fig S2.pptx]

## Slide 1
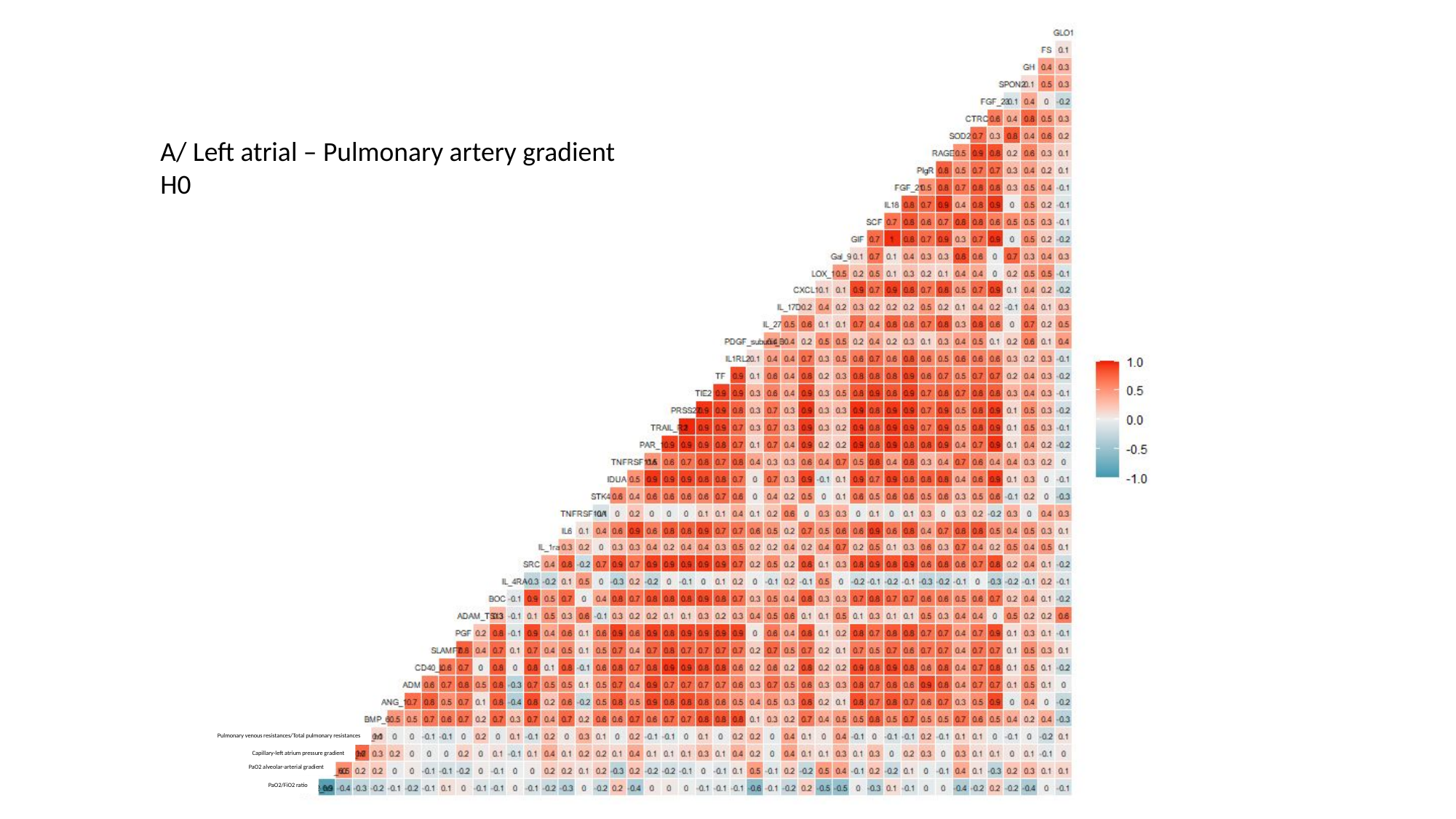

A/ Left atrial – Pulmonary artery gradient
H0
Pulmonary venous resistances/Total pulmonary resistances
Capillary-left atrium pressure gradient
PaO2 alveolar-arterial gradient
PaO2/FiO2 ratio

## Slide 2
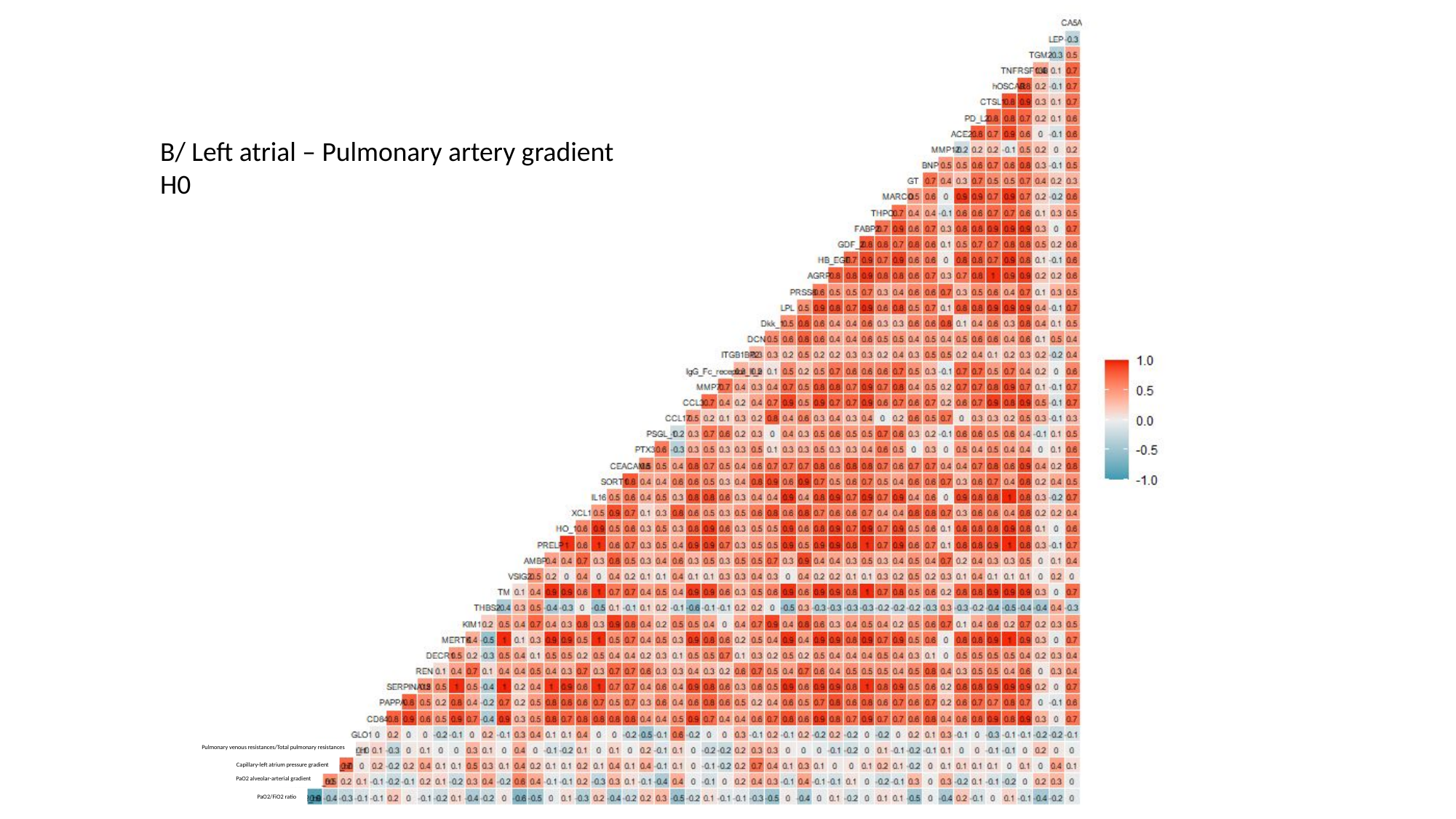

B/ Left atrial – Pulmonary artery gradient
H0
Pulmonary venous resistances/Total pulmonary resistances
Capillary-left atrium pressure gradient
PaO2 alveolar-arterial gradient
PaO2/FiO2 ratio

## Slide 3
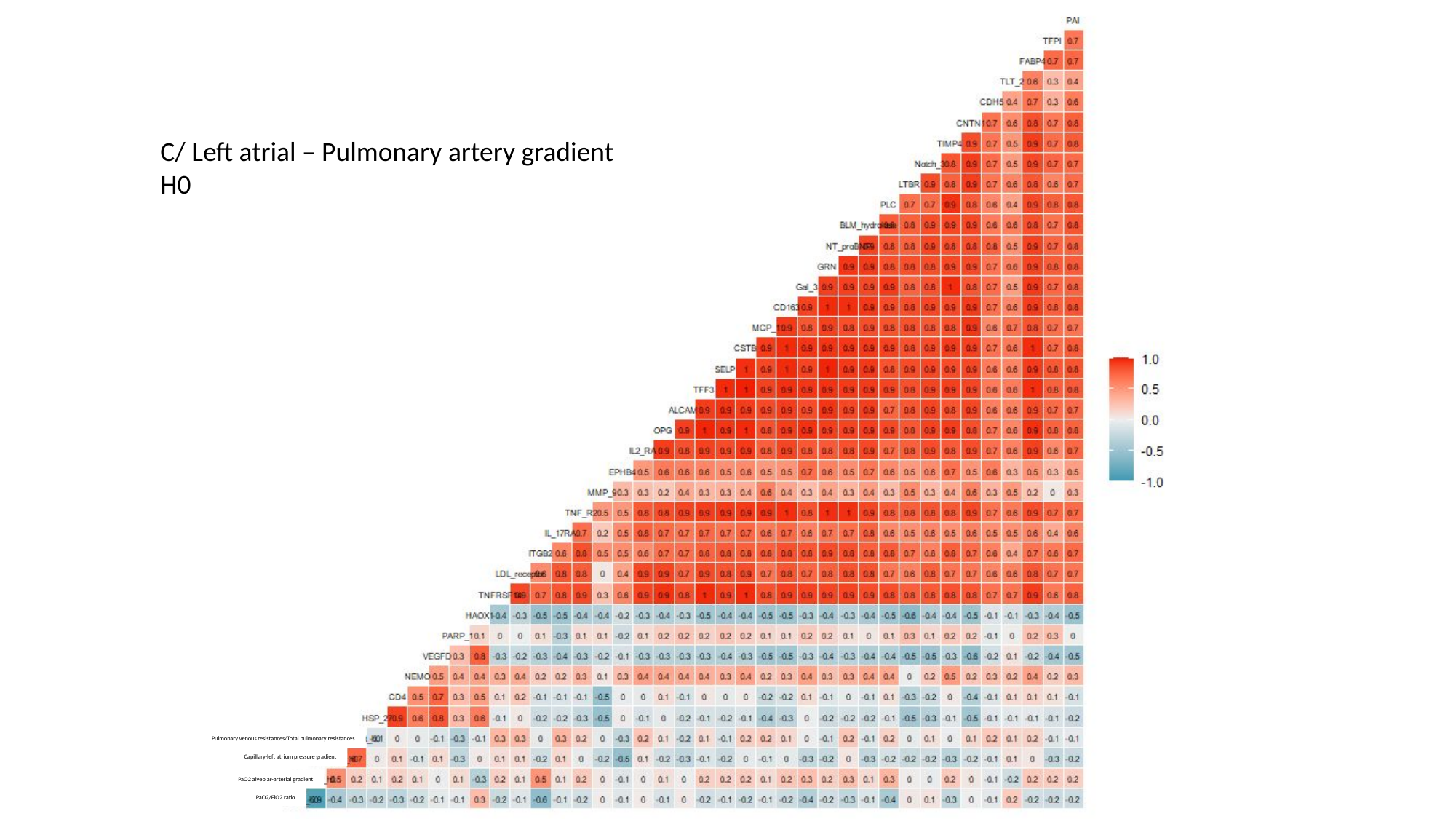

C/ Left atrial – Pulmonary artery gradient
H0
Pulmonary venous resistances/Total pulmonary resistances
Capillary-left atrium pressure gradient
PaO2 alveolar-arterial gradient
PaO2/FiO2 ratio

## Slide 4
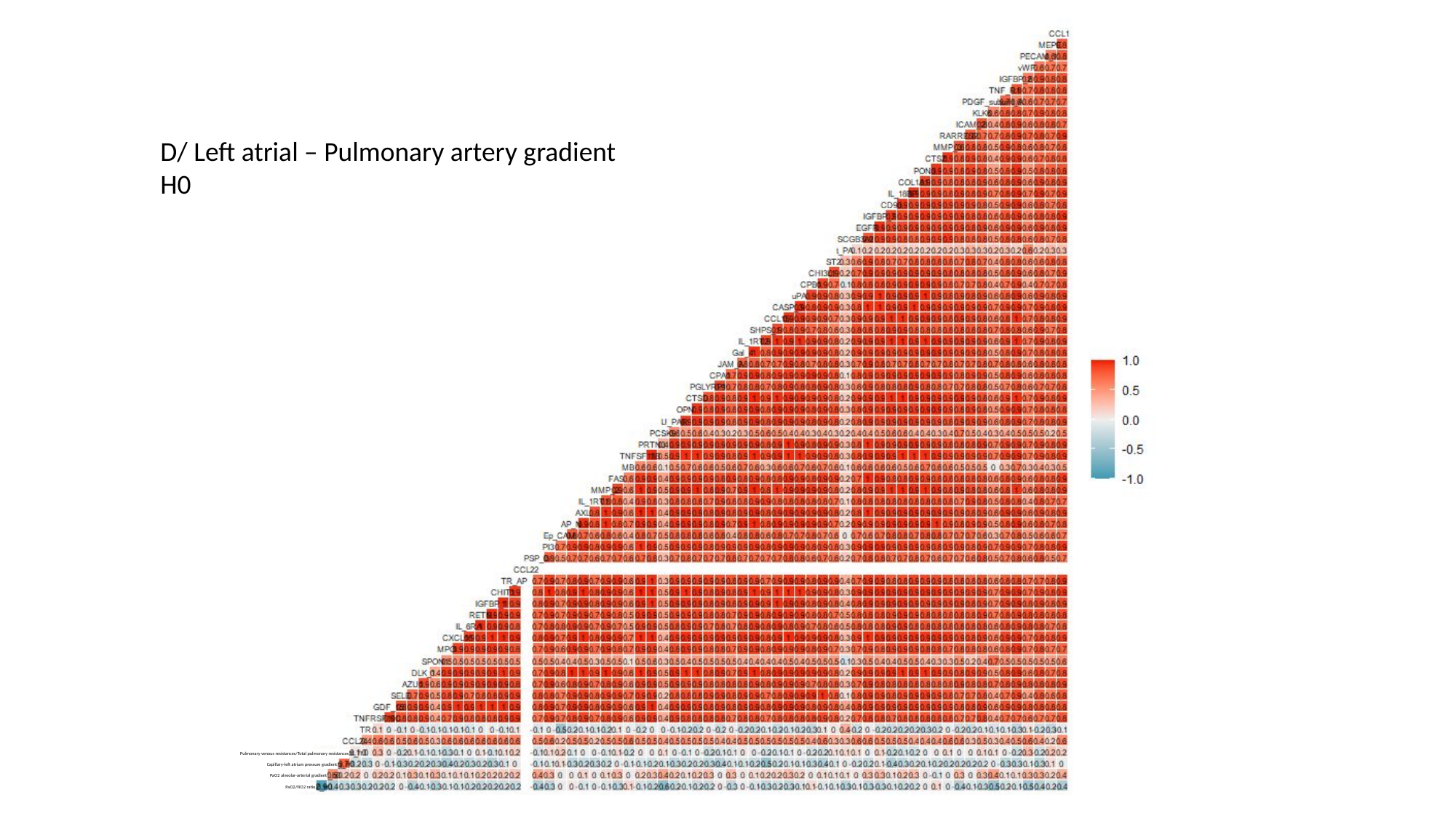

D/ Left atrial – Pulmonary artery gradient
H0
Pulmonary venous resistances/Total pulmonary resistances
Capillary-left atrium pressure gradient
PaO2 alveolar-arterial gradient
PaO2/FiO2 ratio

## Slide 5
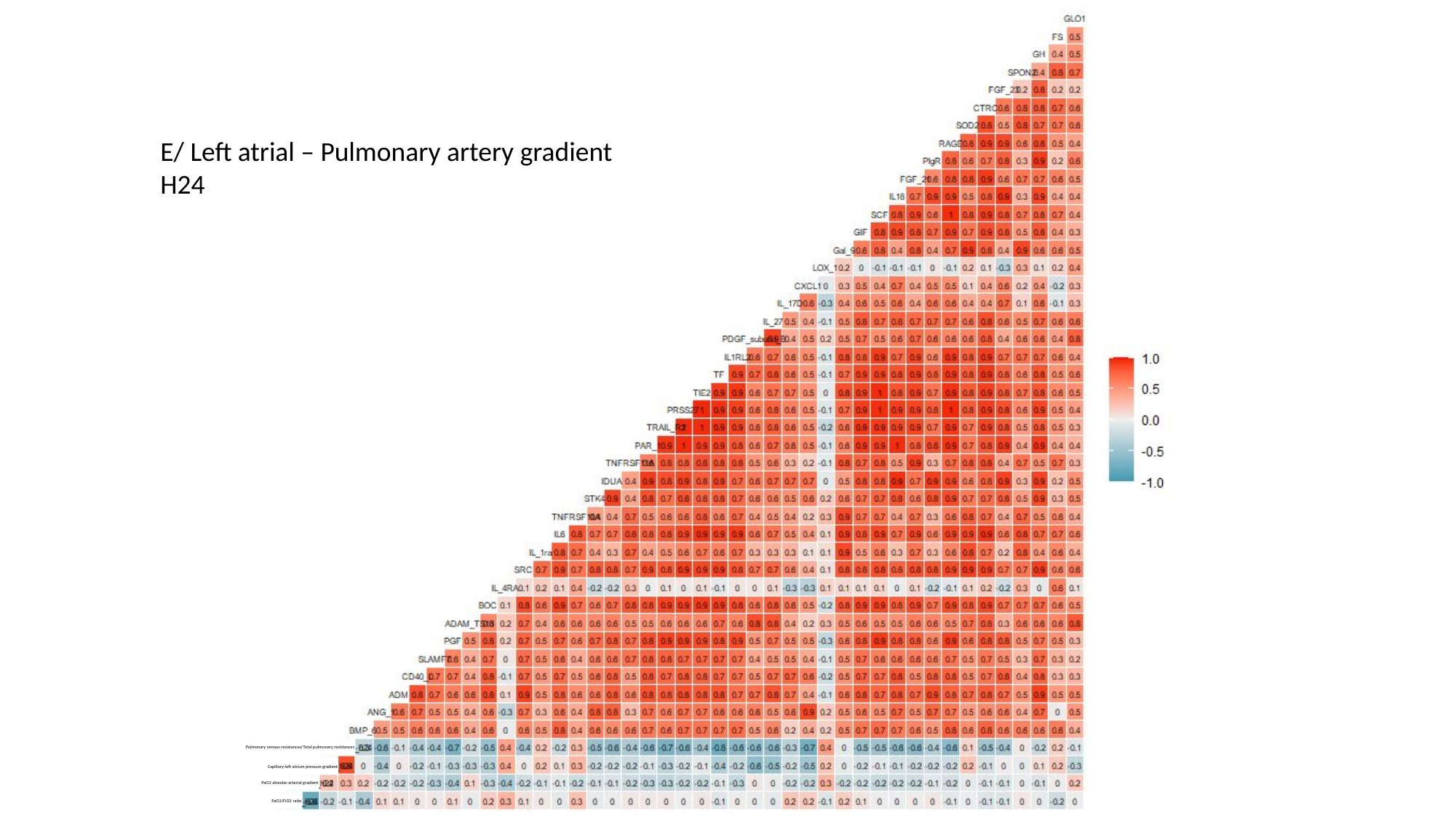

E/ Left atrial – Pulmonary artery gradient
H24
Pulmonary venous resistances/Total pulmonary resistances
Capillary-left atrium pressure gradient
PaO2 alveolar-arterial gradient
PaO2/FiO2 ratio

## Slide 6
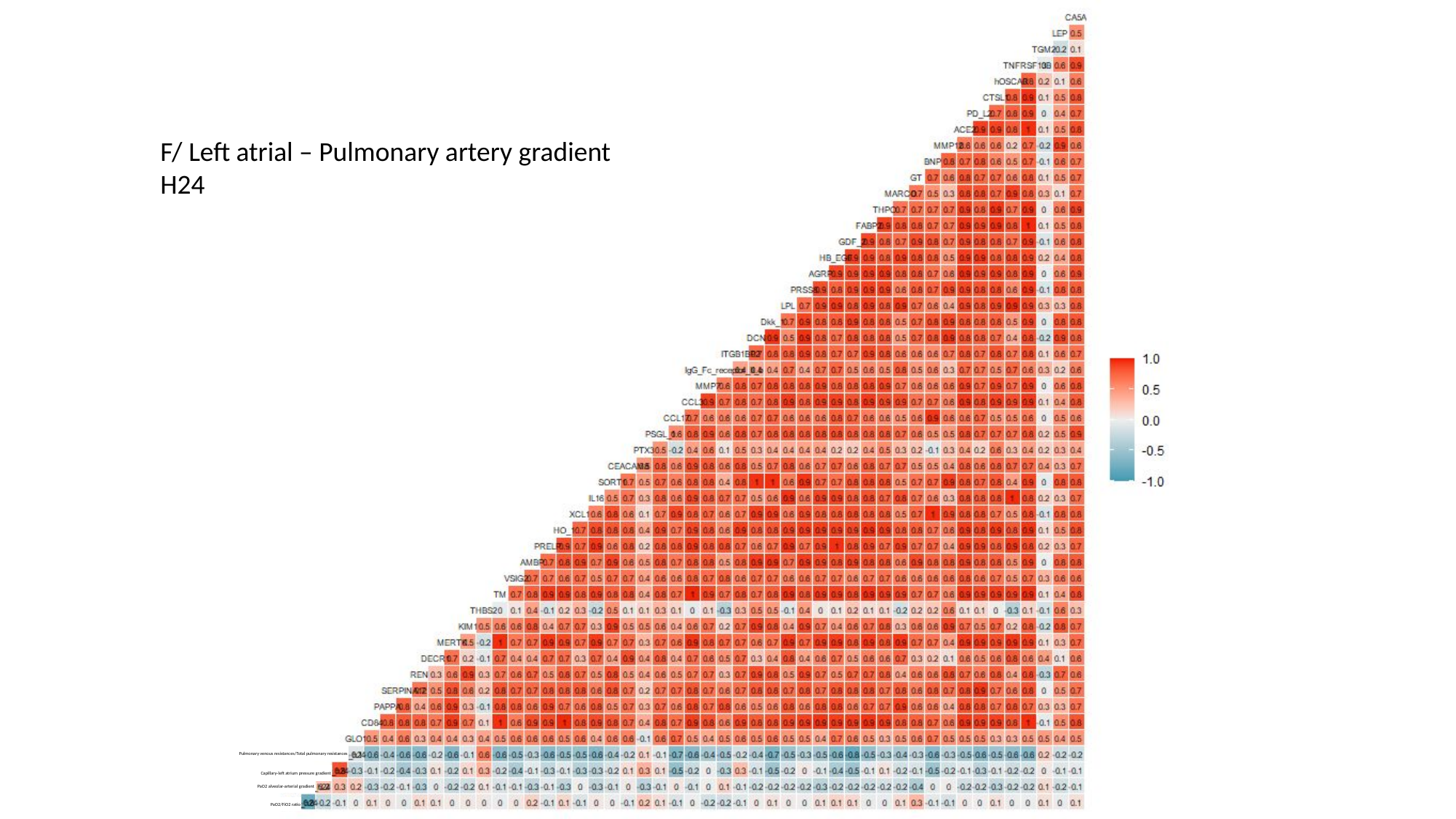

F/ Left atrial – Pulmonary artery gradient
H24
Pulmonary venous resistances/Total pulmonary resistances
Capillary-left atrium pressure gradient
PaO2 alveolar-arterial gradient
PaO2/FiO2 ratio

## Slide 7
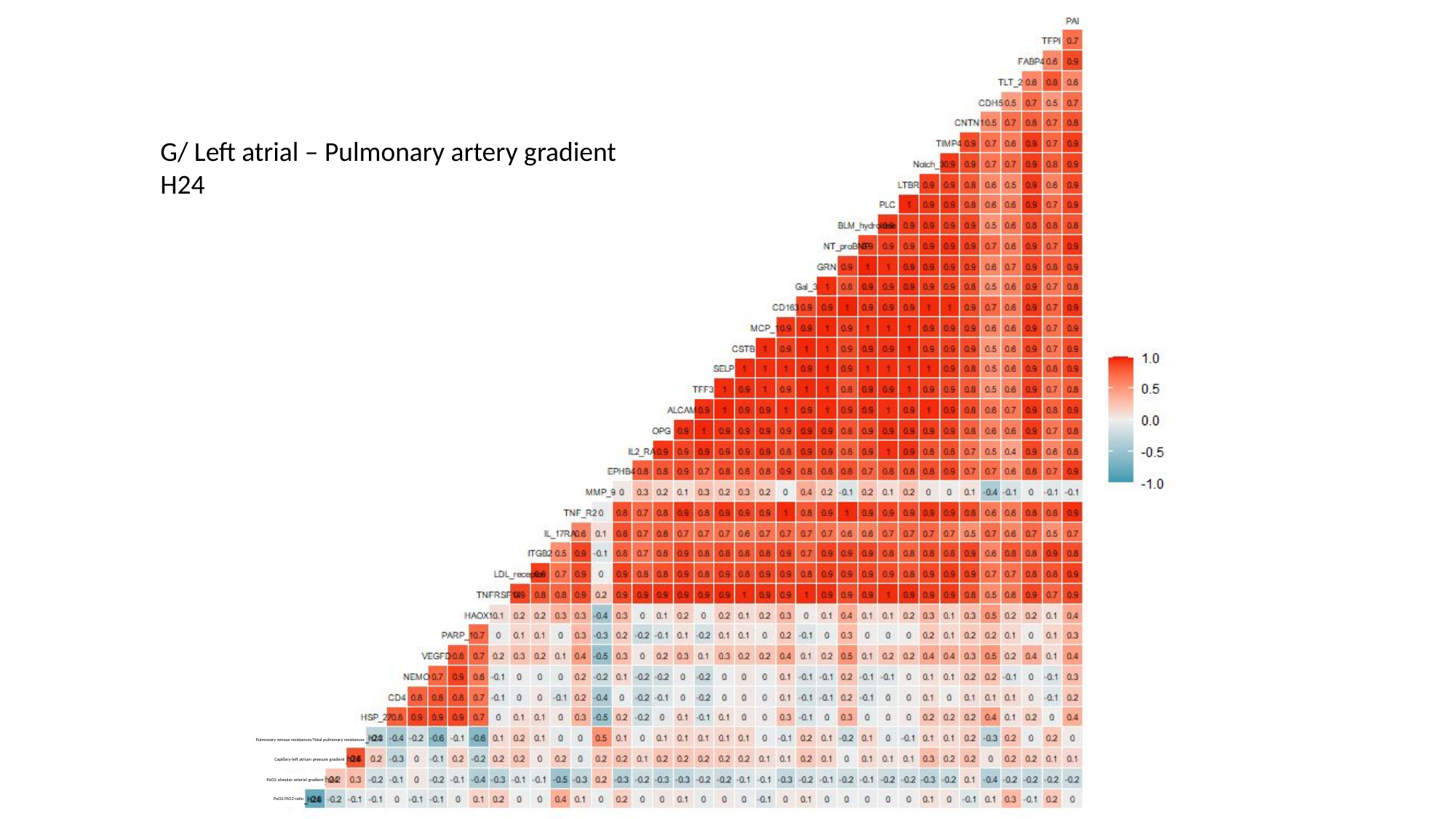

G/ Left atrial – Pulmonary artery gradient
H24
Pulmonary venous resistances/Total pulmonary resistances
Capillary-left atrium pressure gradient
PaO2 alveolar-arterial gradient
PaO2/FiO2 ratio

## Slide 8
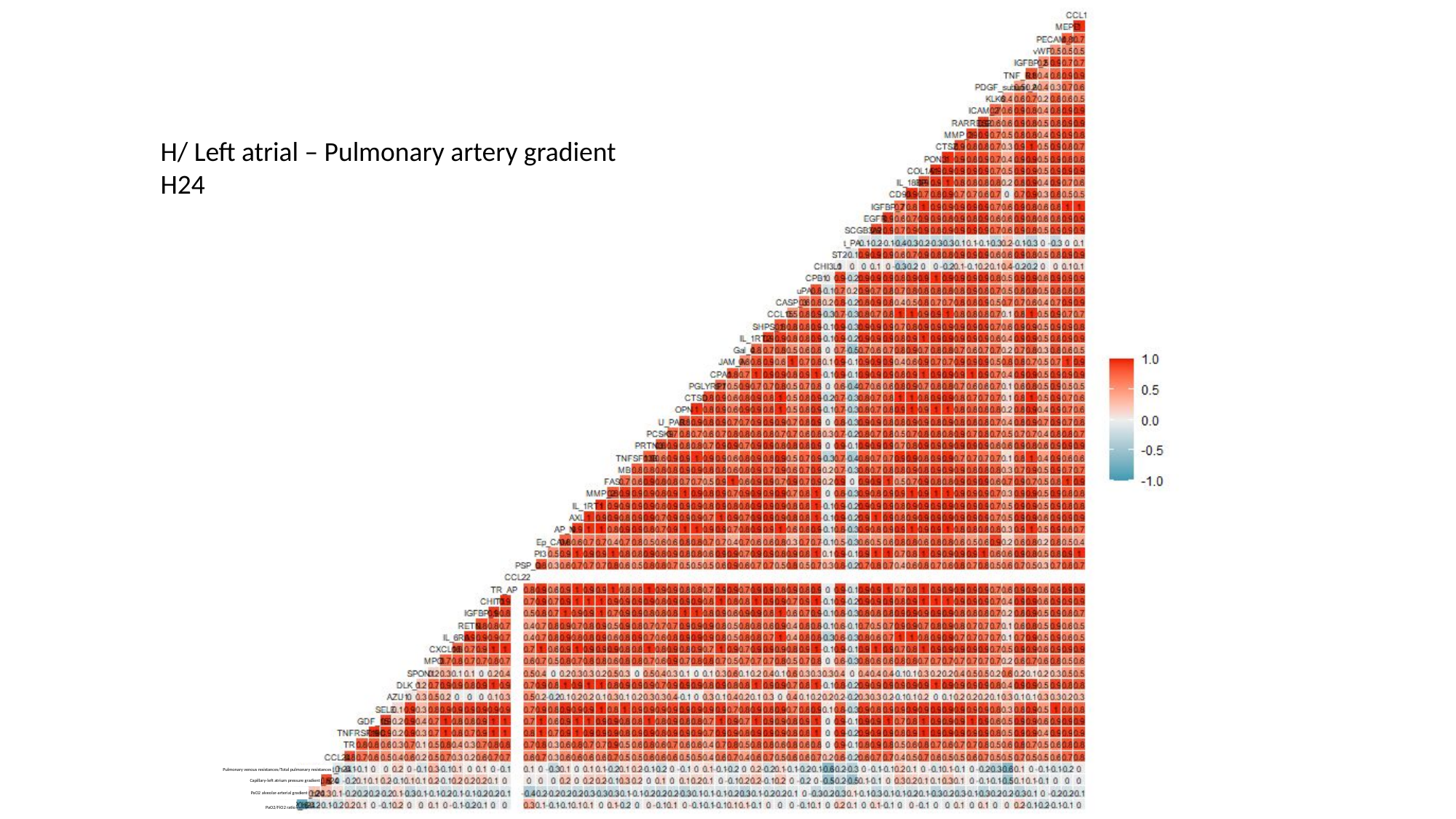

H/ Left atrial – Pulmonary artery gradient
H24
Pulmonary venous resistances/Total pulmonary resistances
Capillary-left atrium pressure gradient
PaO2 alveolar-arterial gradient
PaO2/FiO2 ratio
