# Supplementary figures and images for: Transpulmonary LOX-1 Levels Are Predictive of Acute Respiratory Distress Syndrome After Cardiac Surgery: A Proof-of-Concept Study
Source: Biomedicines. 2025 Mar 26;13(4):800. doi: 10.3390/biomedicines13040800 (PMC12024757; doi:10.3390/biomedicines13040800)

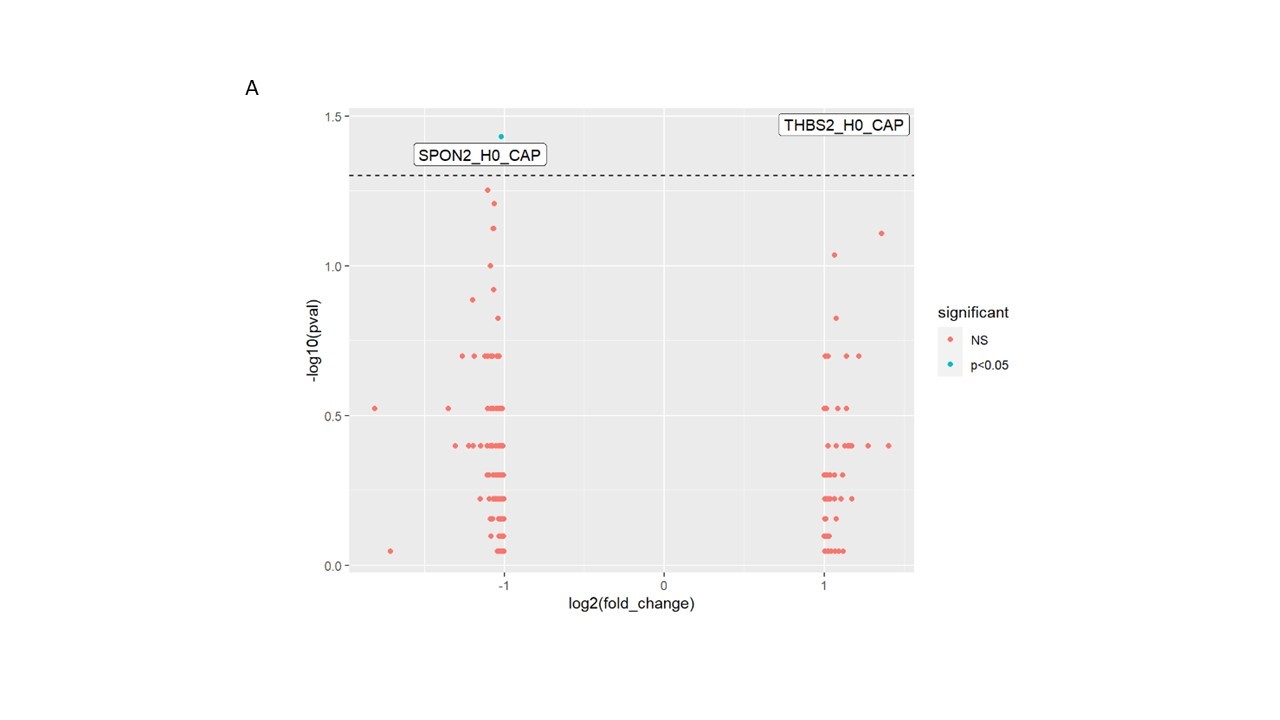

Supplement: Supplementary file 1 [file biomedicines-13-00800-s001.zip › Figure S1_A.jpg]

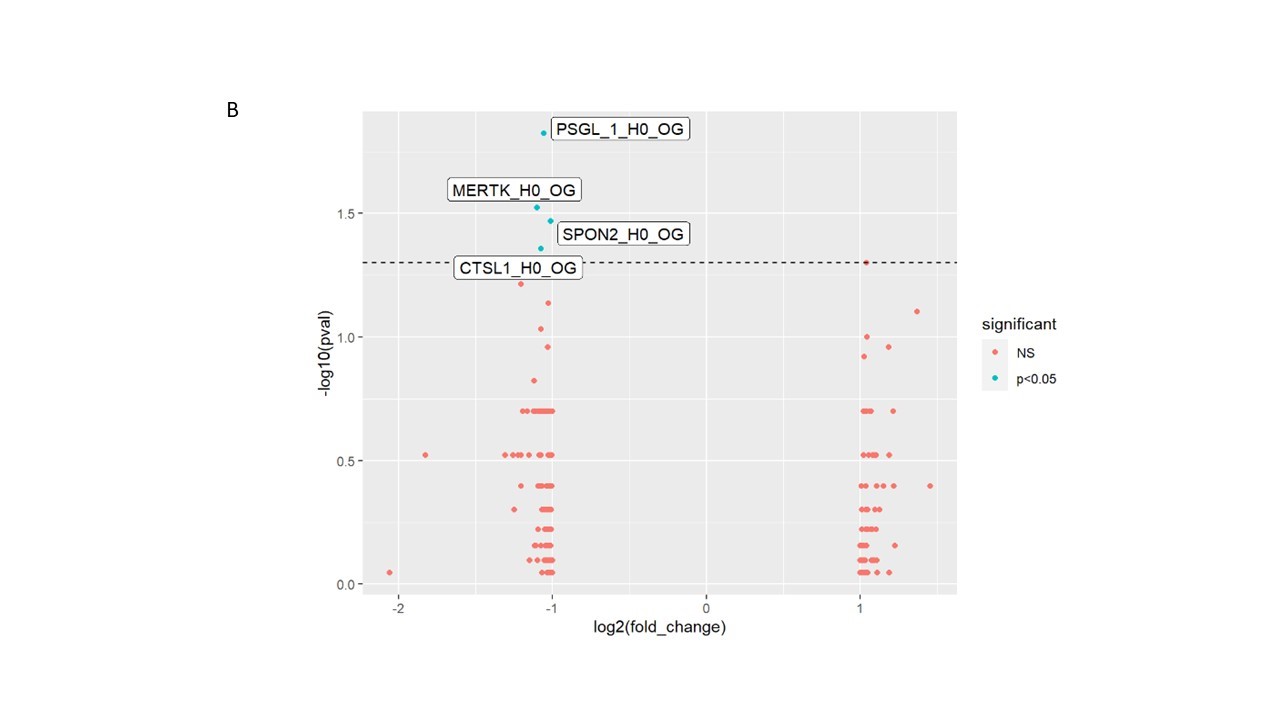

Supplement: Supplementary file 1 [file biomedicines-13-00800-s001.zip › Figure S1_B.jpg]

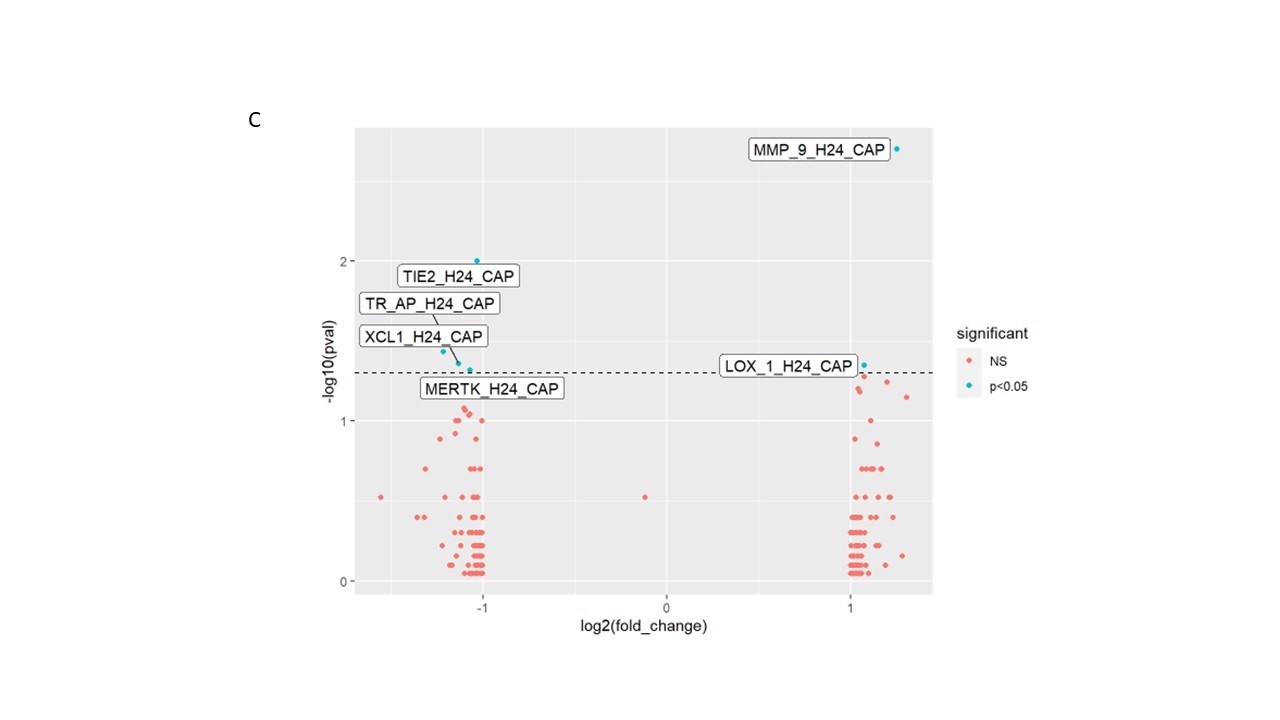

Supplement: Supplementary file 1 [file biomedicines-13-00800-s001.zip › Figure S1_C.jpg]

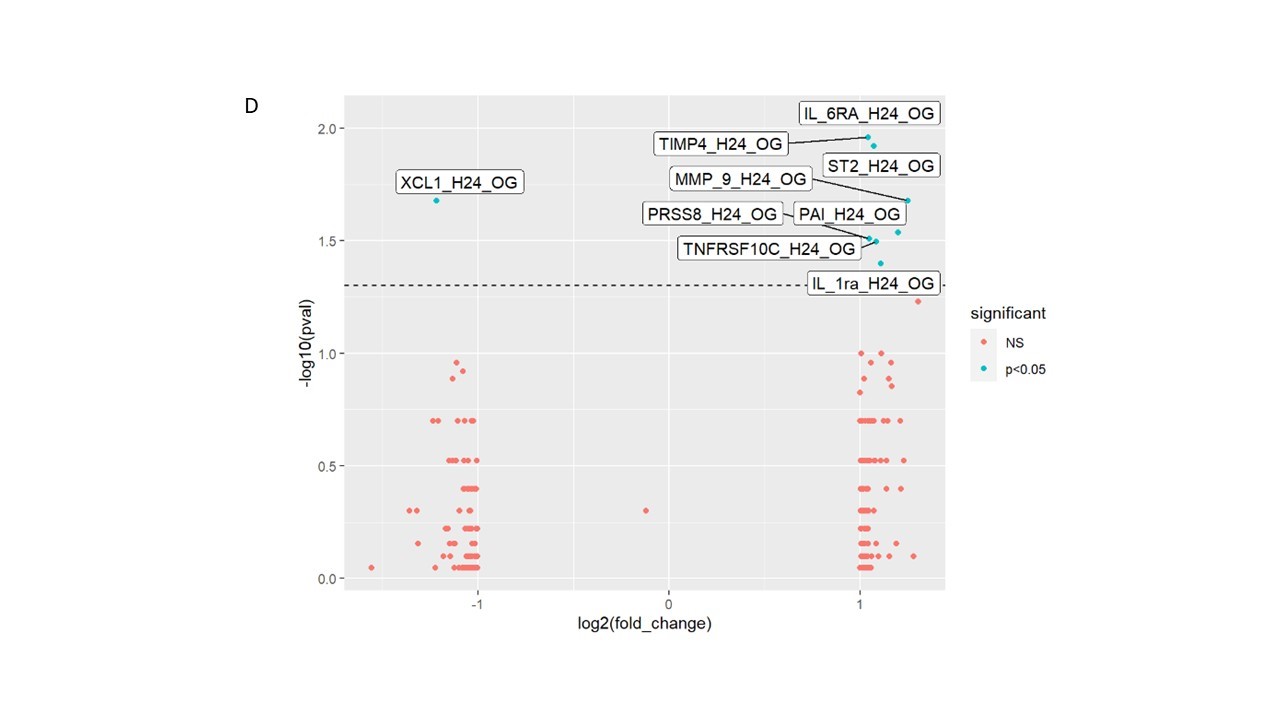

Supplement: Supplementary file 1 [file biomedicines-13-00800-s001.zip › Figure S1_D.jpg]

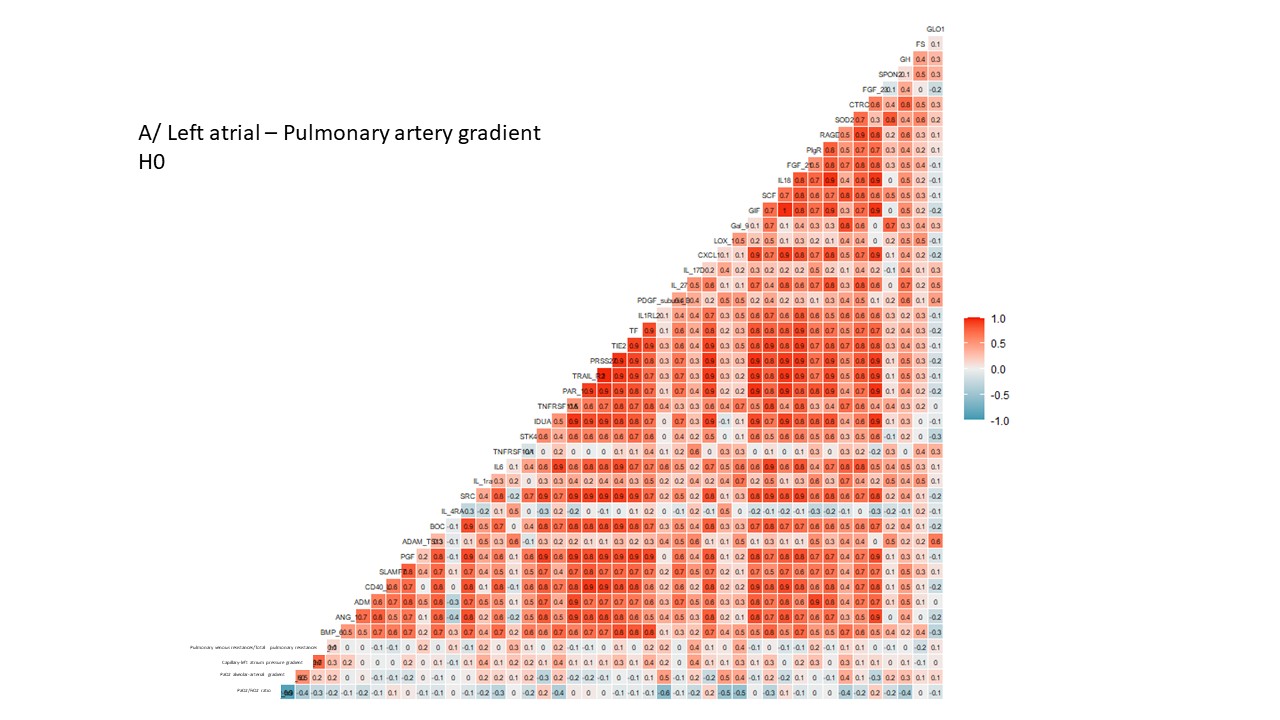

Supplement: Supplementary file 1 [file biomedicines-13-00800-s001.zip › Supplementary Fig S2A.jpg]

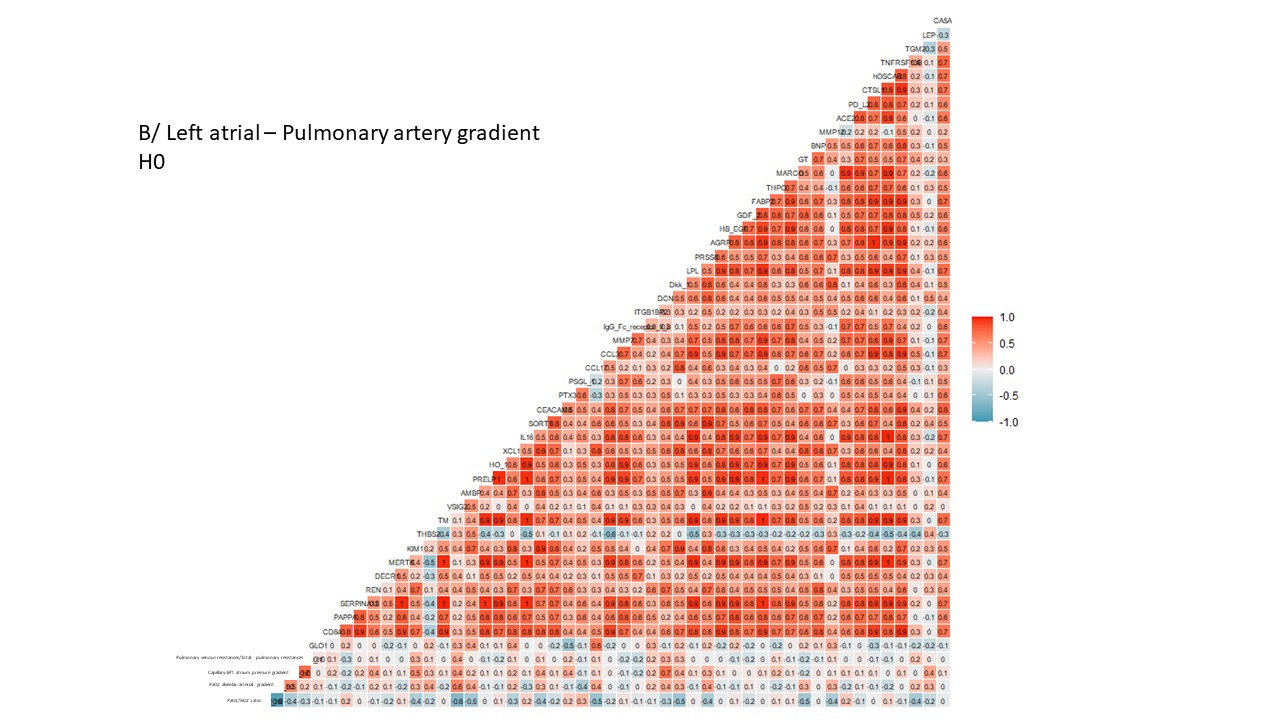

Supplement: Supplementary file 1 [file biomedicines-13-00800-s001.zip › Supplementary Fig S2B.jpg]

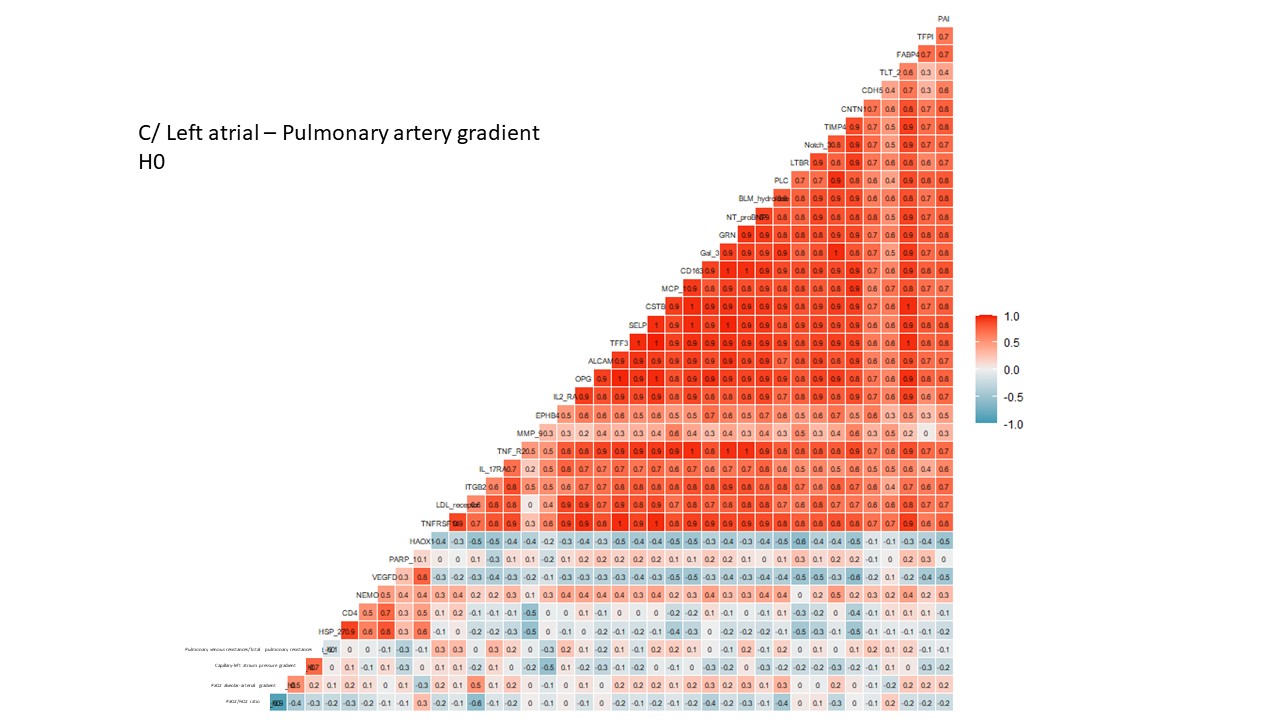

Supplement: Supplementary file 1 [file biomedicines-13-00800-s001.zip › Supplementary Fig S2C.jpg]

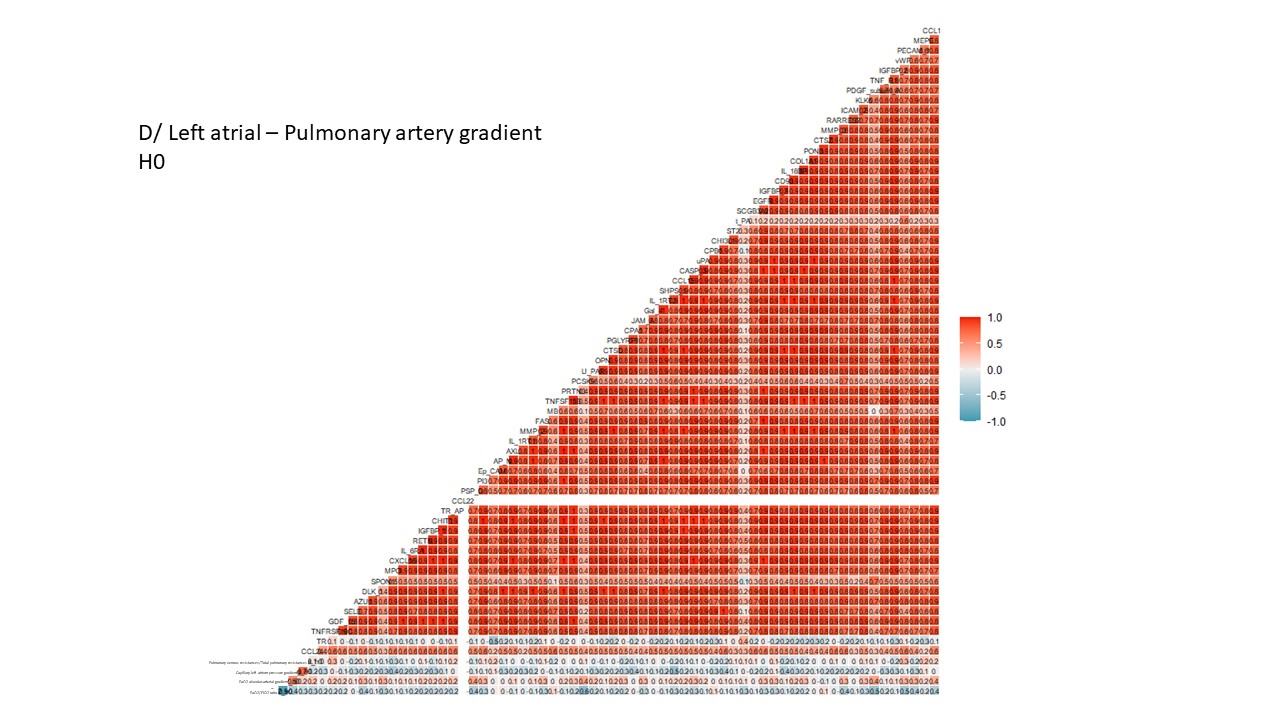

Supplement: Supplementary file 1 [file biomedicines-13-00800-s001.zip › Supplementary Fig S2D.jpg]

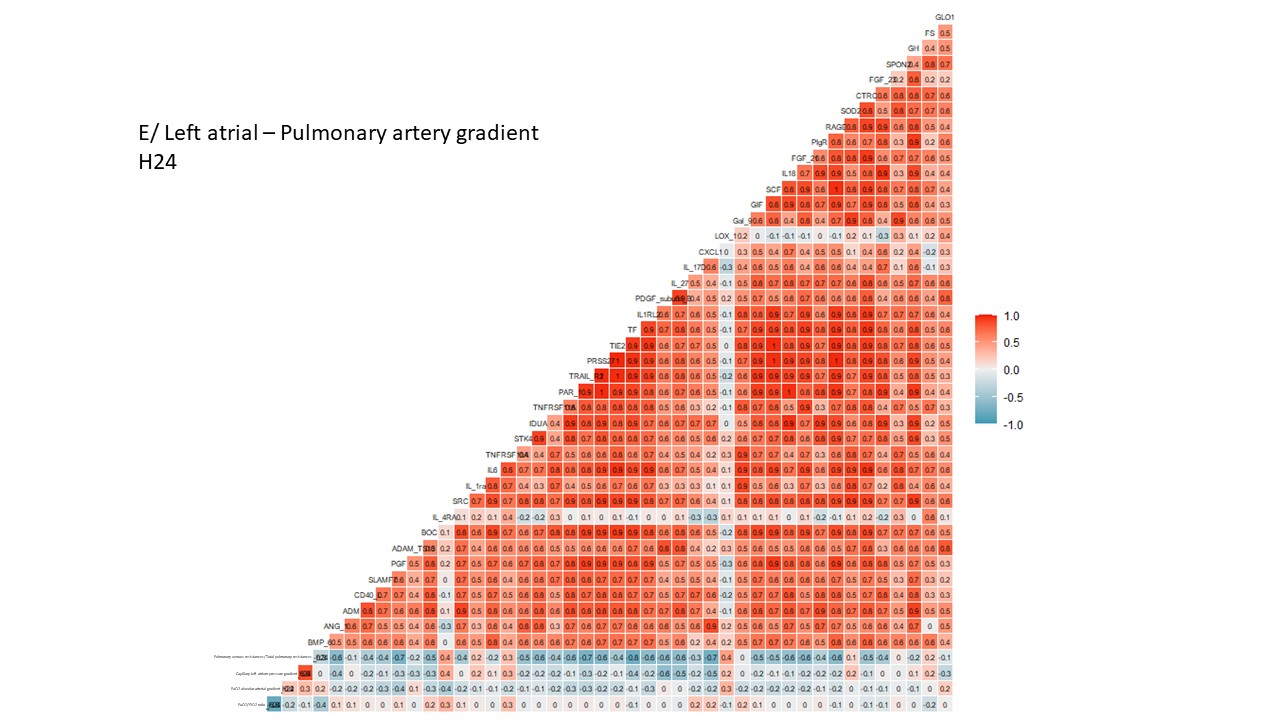

Supplement: Supplementary file 1 [file biomedicines-13-00800-s001.zip › Supplementary Fig S2E.jpg]

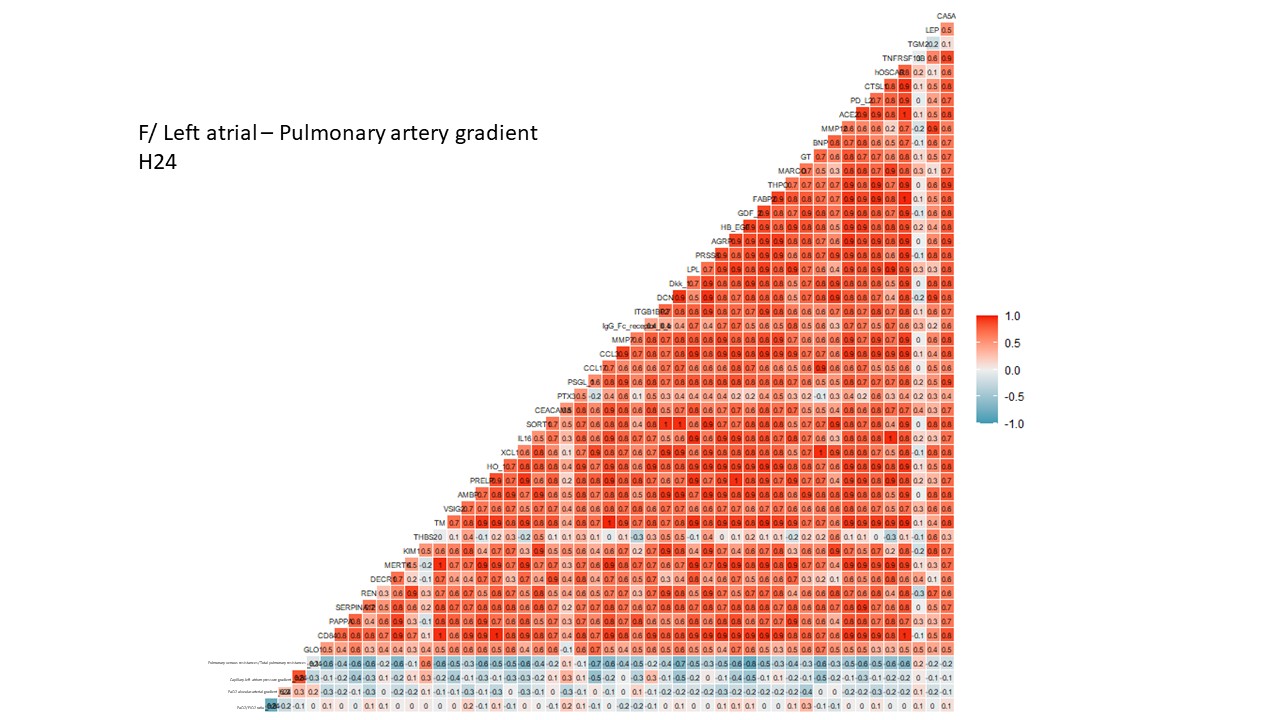

Supplement: Supplementary file 1 [file biomedicines-13-00800-s001.zip › Supplementary Fig S2F.jpg]

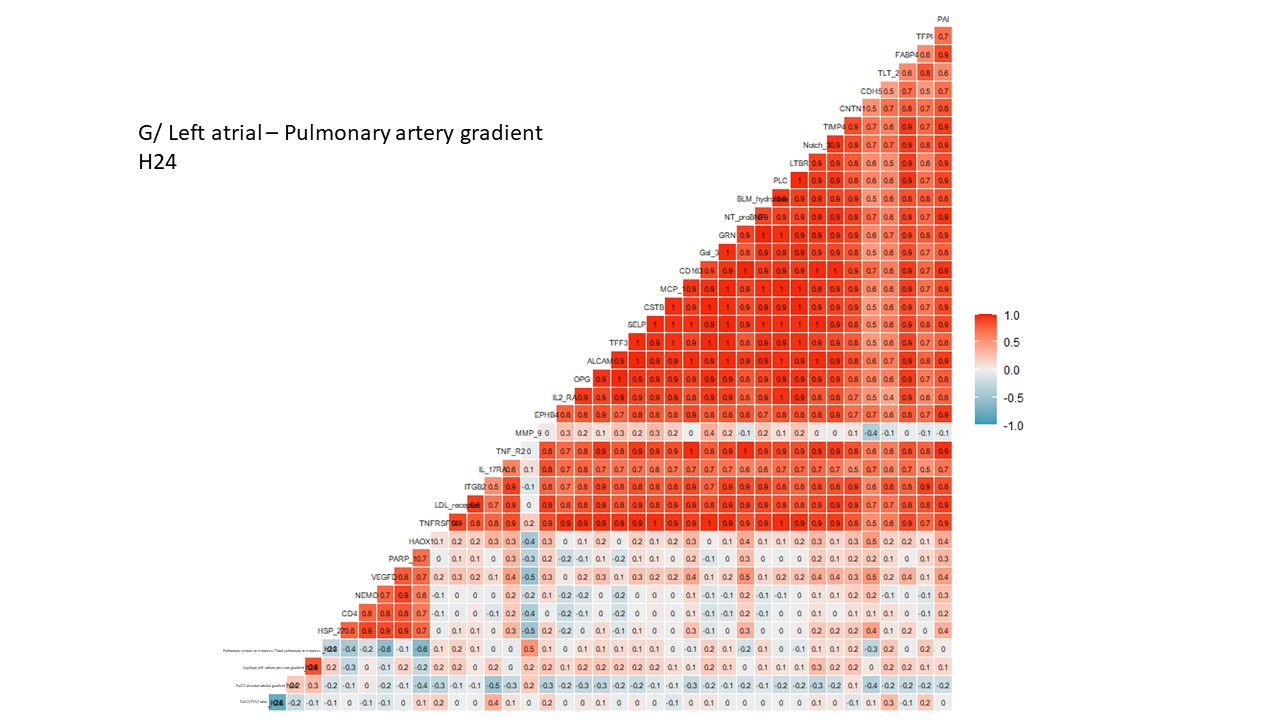

Supplement: Supplementary file 1 [file biomedicines-13-00800-s001.zip › Supplementary Fig S2G.jpg]

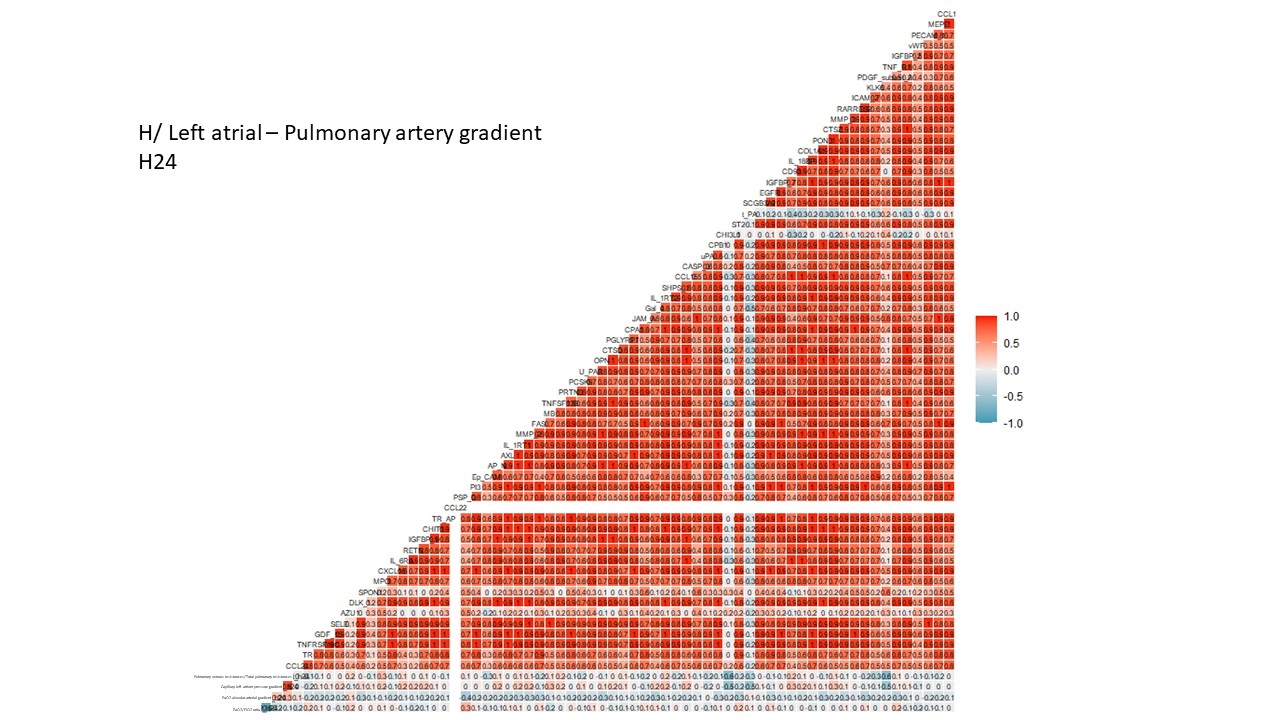

Supplement: Supplementary file 1 [file biomedicines-13-00800-s001.zip › Supplementary Fig S2H.jpg]
